# Related donor platelet transfusion improves platelet transfusion refractoriness in hematological patients

**DOI:** 10.3389/fmed.2023.983644

**Published:** 2023-03-01

**Authors:** Jing-Cheng Zhang, Li-Hong Ni, Yan Tu, Hui-Xian Hu

**Affiliations:** Department of Hematology, Affiliated Jinhua Hospital, Zhejiang University School of Medicine, Jinhua, China

**Keywords:** platelet refractoriness, transfusion, human leukocyte antigens, blood platelets, relative donor platelet

## Abstract

**Objective:**

Transfusion of HLA-matched platelets can reduce the effect of alloimmune responses on platelet transfusion efficacy; however, finding HLA-matched platelets in the population is nearly impossible. Almost all HLA-matched platelets from related are half-matched, but the hemostatic efficacy of related donor platelets is unclear. Our goal was to compare the hemostatic effect of related donated platelets and unrelated donors platelets.

**Methods:**

In this retrospective cohort study, we included acute leukemia and myelodysplastic syndrome patients with thrombocytopenia after chemotherapy. These patients were all transfused with platelets. This study excluded patients younger than 16 years and older than 65 years, or patients with abnormal coagulation parameters during platelet transfusion. We compared the hemostatic effect of related donated platelets and unrelated donors platelet. The primary outcome was transfusion efficacy after platelet transfusion, and the number of platelet counts and corrected count increments at 24 h after platelet transfusion.

**Result:**

We analyzed 31 patients who received platelet transfusions from related donors (Treatment group) and 35 patients who received platelet transfusions from unrelated donors (Comparator group). Except for the relatively small proportion of patients with myelodysplastic syndrome in the treatment group, baseline clinical and laboratory characteristics were similar between the two groups. Hemostasis and prevention of bleeding in the treatment group showed significant superiority; the number of platelets increased 24 h after platelet transfusion in the treatment group was significantly higher than that in the comparator group. After 24 h, the corrected count increments treatment group was also higher than the comparator group; in the treatment group, the transfusion effect was better when the three sites of HLA-A, B, and C were identical, and the different blood types of platelet donors and recipients did not affect the transfusion effect.

**Conclusion:**

Related donated platelets have better hemostasis and prevention effects, and no increase in adverse blood transfusion reactions. It may be a better transfusion strategy for platelet refractoriness patients in emergency situations.

## Introduction

Platelet transfusions are a common practice for preventing or treating bleeding in patients with thrombocytopenia, and approximately 2 million transfusions of platelet components are performed each year in the United States (1). However, repeated platelet transfusions can lead to challenging platelet refractoriness (PTR) (2), patients with PTR have poorer outcomes, and hospitalization costs are three times higher than those without PTR (3). Corrected count increment (CCI) and percentage platelet recovery (PPR) are the most commonly used indicators to evaluate the effect of platelet transfusion. It is generally believed that patients with CCI <7.5 × 10^9^/L 1 h after transfusion of ABO isotype platelets, CCI <7.5 × 109/L at 24 h, or PPR <20% at 24 h of platelet transfusion can be judged as PTR (4, 5).

Both immune and non-immune factors can cause PTR, with non-immune factors usually accounting for 60–80% (6). However, in patients with hematological malignancies and severe aplastic anemia (SAA) requiring multiple platelet transfusions, the formation of human leukocyte antigen (HLA) alloimmune antibodies is a major factor leading to PTR (7). HLA antigens are present both in leukocytes and on the surface of platelets. However, only HLA-I antigens are expressed on the platelet membrane, mainly HLA-A and HLA-B antigens, and only a small amount of HLA-C antigens (8). The clinical response to HLA alloimmunization ineffective platelet transfusion is to select HLA-matched donor platelets. However, this strategy is labor-intensive and requires maintaining a large pool of available HLA-type apheresis donors, such as approximately 12,000 donors in England to match HLA-matched donor platelets (9). The proportion of all HLA-identical sibs in Chinese siblings reaches 25%, and HLA class I antigens are approximately 80% identical in sibs of parents and children (10). However, data on whether platelet transfusions donated by family members can improve transfusion outcomes are lacking.

We conducted a retrospective cohort study to assess the hemostatic and bleeding prevention effects of platelets donation from related and from unrelated donors. As a secondary outcome, we examined HLA-A, B, and C antibodies in blood platelets from related sources to determine whether HLA-class I-matched platelets could reduce PTR. We also assessed the incidence of transfusion-associated graft versus host disease (TA-GvHD) and other adverse effects of platelet transfusions donated by family members.

## Materials and methods

### Study design and participants

This single-center, retrospective cohort study included patients with acute leukemia (AL) and myelodysplastic syndrome (MDS) diagnosed at Affiliated Jinhua Hospital, Zhejiang University School of Medicine, between 20 October 2016 and 20 November 2021. Eligible patients were between the ages of 16 and 65 years and with platelets less than 20 × 10^9^/L requiring platelet transfusion therapy. Patients with palpable splenomegaly, abnormal coagulation function before platelet transfusion, body temperature above 38°C, and etiologically proven infection were excluded from this study.

The study was approved by the Affiliated Jinhua Hospital, Zhejiang University School of Medicine (JH-2021-12-01) to allow retrospective access to patients’ records and files.

### Platelet products and transfusion policy

Related donor platelets include the patient’s immediate siblings, parents, or children. Platelets were obtained by separating whole blood from Spectra Optia Blood Cell Separator (Carlsbad, CA, United States), and the number of platelets in each bag was ≥2.5 × 10^11^/L, and were transfused within 24 h on the day of collection or stored at 4°C. Unrelated platelets were provided by Jinhua Blood Center and met the requirements of Chinese quality standards (11). Each bag platelets is 9–12 units, and the number of platelets is ≥2.5 × 10^11^/L. They are stored in a shaking box at 22°C, and they are transfused within the validity period. Most of the platelets in the two groups were transfused prophylactically (triggered by platelet counts less than 20 × 10^9^/L) and a small number were transfused therapeutically. According to China’s platelet transfusion policy, platelet transfusion from the central blood bank is blood type matching (control group), and only platelet donation from family members (treatment group) has blood type incompatibility. At the same time, the need for transfusion of red blood cell concentrate and plasma is determined according to the blood transfusion threshold guided by the hospital and the judgment of the treating physician.

### Outcomes and clinical assessments

The primary outcome was the percentage of patients with a World Health Organization (WHO) grade 1, 2, 3, or 4 bleeding event. The evaluation period was 7 days after the patient’s first platelet transfusion after chemotherapy. Daily bleeding assessment forms were filled out by trained staff on a daily basis, and all written descriptions of bleeding were reviewed by two assessors unaware of the treatment assignment to minimize bias.

Secondary outcomes were increased platelet counts and CCI 24 h after platelet transfusion, the number of patients who developed PRT, and transfusion-related adverse reactions. In the treatment group, whether the transfusion of different blood types and HLA class I antigen-matched platelets increased 24-h CCI.

### Statistical analysis

We used χ^2^ and Fisher’s exact test for categorical variables, and t-test for continuous variables to assess the difference in means between the two groups. A two-sided α of less than 0.05 was considered statistically significant. Statistical analyzes were done using the SPSS Statistics software (version 22) and GraphPad Prism (version 9), unless otherwise indicated.

## Results

### Study population

**Figure 1:**
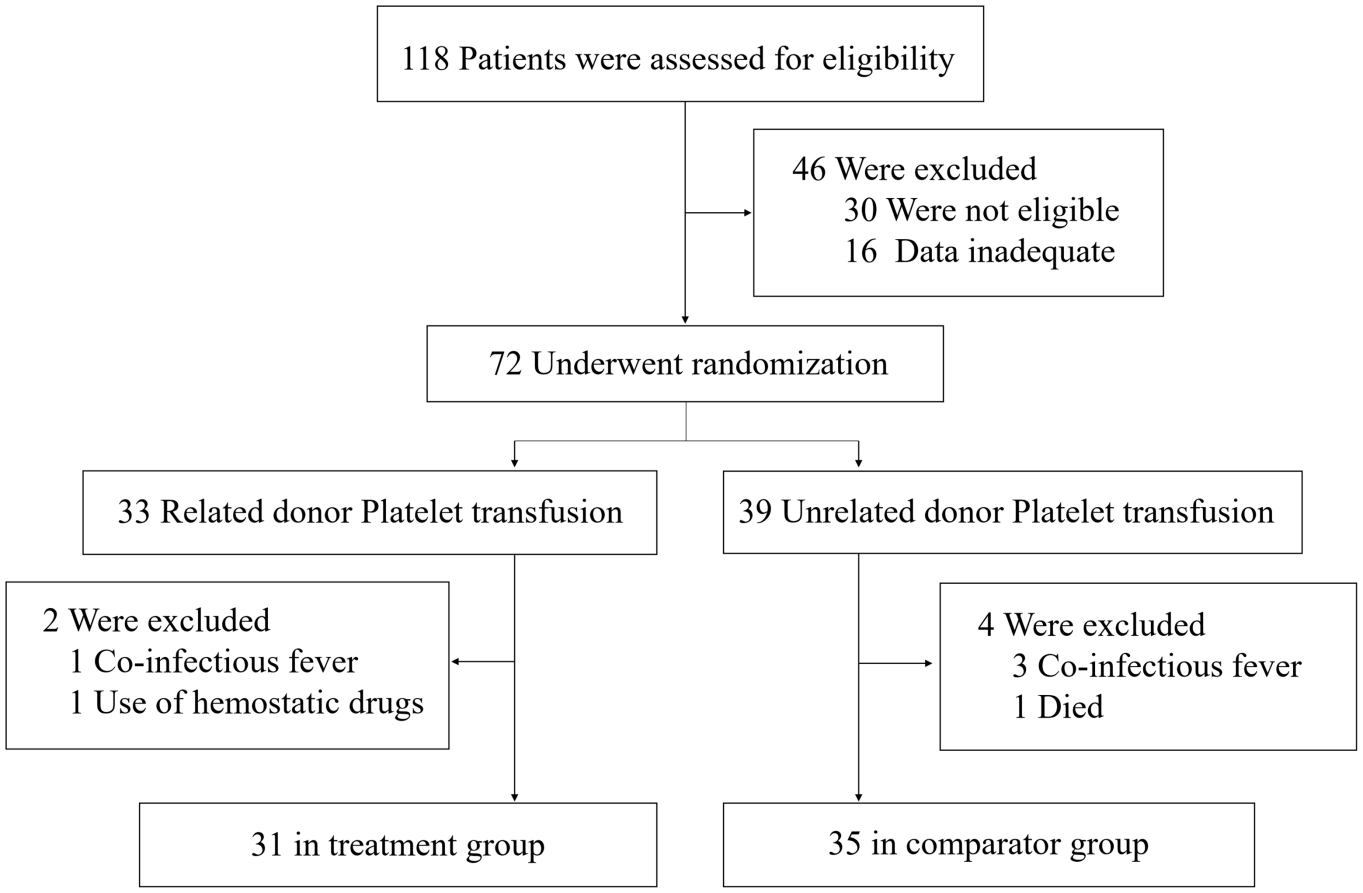
Study profile.

Between 20 October 2016 and 20 November 2021, 118 people were diagnosed with AL or MDS and had platelet counts<20 × 10^9^/L. The clinical baseline characteristics of patients with related donors platelet transfusion (Treatment group) and unrelated donor platelet transfusion (Comparator group) were generally similar (Table 1). After excluding ineligible patients, 66 patients were included in the study: 31 in the treatment group and 35 in the comparator group. The cohort construction flowchart is shown in Figure 1.

**Table 1:** Patient characteristics.

	Treatment group (*n* = 31)	Comparator group (*n* = 35)
Male/female, *n*	16/15	19/16
Age, mean ± SD, y	47 ± 16	51 ± 19
Body surface area, mean ± SD, m^2^	1.71 ± 0.52	1.69 ± 0.49
Diagnosis, *n* (%)		
Acute myelogenous leukemia	13 (41.9)	16 (45.7)
Acute lymphoblastic leukemia	16 (51.6)	12 (34.3)
Myelodysplastic syndromes	2 (6.5)^†^	7 (20.0)
Laboratory values at randomization, mean ± SD		
Platelet count, 10^9^/L	13 ± 7	12 ± 8
Hemoglobin, g/L	89 ± 22	84 ± 19
White cell count, 10^9^/L	1.9 ± 0.4	1.8 ± 0.6
Activated partial thromboplastin time, s	31.5 ± 8.9	33.3 ± 9.2
Prothrombin time, s	15.1 ± 3.1	14.9 ± 3.2
Fibrinogen, g/L	3.5 ± 1.9	3.4 ± 2.5
Medication and medical history, *n* (%)		
Anticoagulant/antiplatelet therapy	0 (0)	0 (0)
Bleeding	2 (9.5)	3 (8.6)
Prior platelet transfusion	29 (93.5)	33 (94.3)
Prior red-cell transfusion	31 (100)	35 (100)
Prior pregnancy	14 (45.2)	17 (48.6)
Blood type, *n* (%)		
A	5 (16.1)	7 (20.0)
B	15 (48.4)	16 (45.7)
AB	3 (9.7)	5 (14.3)
O	8 (25.8)	7 (20.0)
Rh+	31 (100)	35 (100)

^†^*p* < 0.05.

### Bleeding assessment

We analyzed 31 patients who received platelet transfusions from related donors (Treatment group) and 35 patients who received platelet transfusions from unrelated donors (Comparator group).

Data on bleeding events during the evaluation period are presented in Table 2. WHO grade 1, 2, 3, or 4 bleeding events occurred in 38.7% of patients in treatment group (12 of 31 patients) compared with 82.9% of patients in arm comparator group (29 of 35 patients). The comparison between the two groups showed a significant superiority of treatment group (*p* = 0.05). Therefore, this study show that treatment group was less than comparator group in terms of the frequency of WHO grade 1, 2, 3, or 4 bleeding events.

**Table 2:** Bleeding complications.^*^

	Treatment group (*n* = 31)	Comparator group (*n* = 35)
Primary endpoint		
WHO grade 1, 2, 3, or 4 bleeding, *n* (%)^‡^	12 (38.7)	29 (82.9)
No. of days form first episode of grade 1, 2, 3,or 4 bleeding, median (IQR)^†^	4 (2–6)	2 (1–7)
No. of days with grade 1, 2, 3,or 4 bleeding, median (IQR)	2 (1–6)	5 (2–7)
WHO grade 3 or 4 bleeding, *n* (%)^†^	1 (3.2)	1 (2.9)
Highest grade of bleeding, *n* (%)		
None or grade 1	25 (80.6)	27 (77.1)
Grade 2	5 (16.1)	7 (20.0)
Grade 3	1 (3.2)	1 (2.9)
Grade 4	0 (0)	0 (0)
Type of cancer		
Acute myelogenous leukemia^‡^	4 (30.8)	13 (81.2)
Acute lymphoblastic leukemia^‡^	6 (37.5)	11 (91.7)
Myelodysplastic syndromes^†^	2 (100)	5 (71.4)

^*^Plus-minus values are means ± SD. In the World Health Organization (WHO) grading system, bleeding episodes are classified as grade 1 (mucous membrane hemorrhage), grade 2 (moderate; red-cell transfusion not needed immediately), grade 3 (severe; requiring red-cell transfusion within 24 h), or grade 4 [debilitating or life-threatening (11, 12)]. ^†^*p* < 0.05. ^‡^*p* < 0.001.

The number of days with WHO grade 1, 2, 3, or 4 bleeding events during the evaluation period was lower in treatment group than in arm comparator group (median 2 days lower). The time to first bleeding episode was significantly longer in treatment group than in comparator group (median 3 days longer). The proportion of patients with WHO grade 2, 3, and 4 bleeding in treatment group was not significantly different from that in comparator group.

### Platelet transfusions

**Figure 2:**
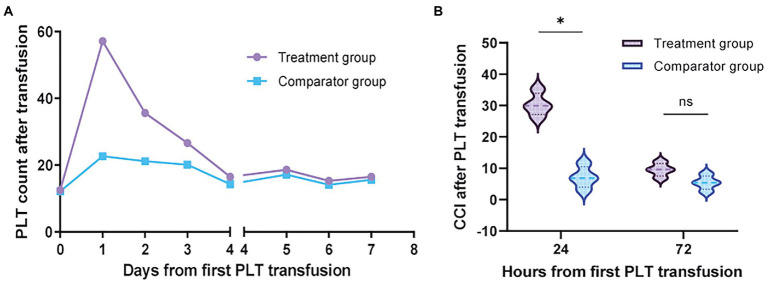
The efficacy of platelet transfusion. **(A)** The increased number of platelets in the peripheral blood of a patient after platelet transfusion. The first platelet transfusion started on d0, and the number of platelets in treatment group was significantly higher than that in comparator group on d1 (*p* < 0.001). On day 4, both groups were transfused with unrelated donor platelets. **(B)** The CCI value of treatment group at 24 h after blood transfusion was significantly higher than that of comparator group (*p* < 0.001). No difference was seen between the two groups after 72 h.

Most platelet transfusions were prophylactic, with no significant differences in platelet counts in the body before platelet transfusion between the two groups and comparable platelet content in transfused products. This study mainly observed the increase in platelet count and CCI at 24 h (range, 14–24 h) and 72 h (range, 52–72 h) after platelet transfusion in the two groups. The specific results are shown in Table 3. The platelet increase in group treatment at 24 h. The amount was significantly higher than that in group comparator (95% confidence interval [CI], 25.9 to 34.9; *p* = 0.001) (Figure 2A). The CCI value of group treatment was also significantly higher than that of group comparator at 24 h [95% confidence interval (CI), 4.0 to 8.2; *p* = 0.001] (Figure 2B). At 72 h, the increased number of platelets and CCI in group treatment were higher than those in group comparator, but there was no significant difference. In addition, group treatment basically did not have PTR, but the proportions of 24-h CCI <10 and PPR <20% in group comparator were 68.5 and 54%, indicating that related donor platelet transfusion can reduce the occurrence of PTR.

**Table 3:** Platelet transfusion increment.

	Treatment group (*n* = 31)	Comparator group (*n* = 35)	*P*
Efficacy parameters, mean ± SD			
Count increment 24 h, 10^9^/L	44.6 ± 10.5	10.5 ± 6.9	<0.001
CCI 24 h	29.8 ± 7.0	7.0 ± 4.6	<0.001
Count increment 72 h, 10^9^/L	14.1 ± 4.1	7.9 ± 4.4	<0.01
CCI 72 h	9.4 ± 2.7	5.3 ± 2.9	<0.05
Transfusion failure rate, *n* (%)			
CCI 24 h, ≤0	0 (0%)	4 (11.4%)	
CCI 24 h, <4.5	0 (0%)	7 (20.0%)	
CCI 24 h, <10	0 (0%)	13 (37.1%)	
PPR 24 h, ≤20%	1 (3.2%)	19 (54.0%)	<0.001

### Safety

**Figure 3:**
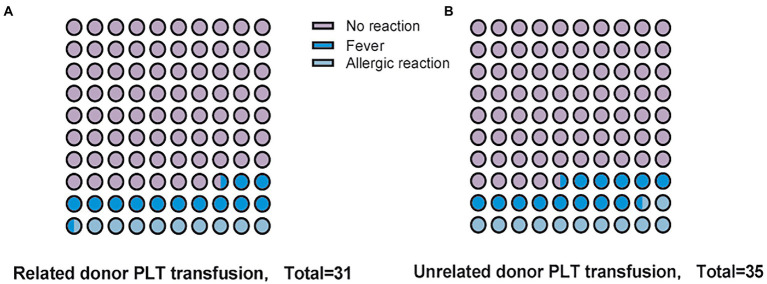
Complications of platelet transfusion. **(A)** Adverse events following transfusion of Related donor platelets. **(B)** Adverse events following transfusion of Unrelated donor platelets.

Common platelet transfusion complications such as fever, anaphylaxis, and hemolysis did not differ between the two groups (Figure 3). There was no event of transfusion-associated graft versus host disease (TA-GVHD) in the treatment group and in the comparator group, and there was 1 case of serious adverse event (SAE) in the treatment group, which was judged by the clinician to be heart failure caused by massive rehydration during platelet transfusion. The percentages of confirmed transfusion adverse event were 19.4 and 25.7% in the treatment and comparator groups. The majority of the transfusion adverse event in both groups resulted in no or only minor morbidity.

### Subgroup analysis

**Figure 4:**
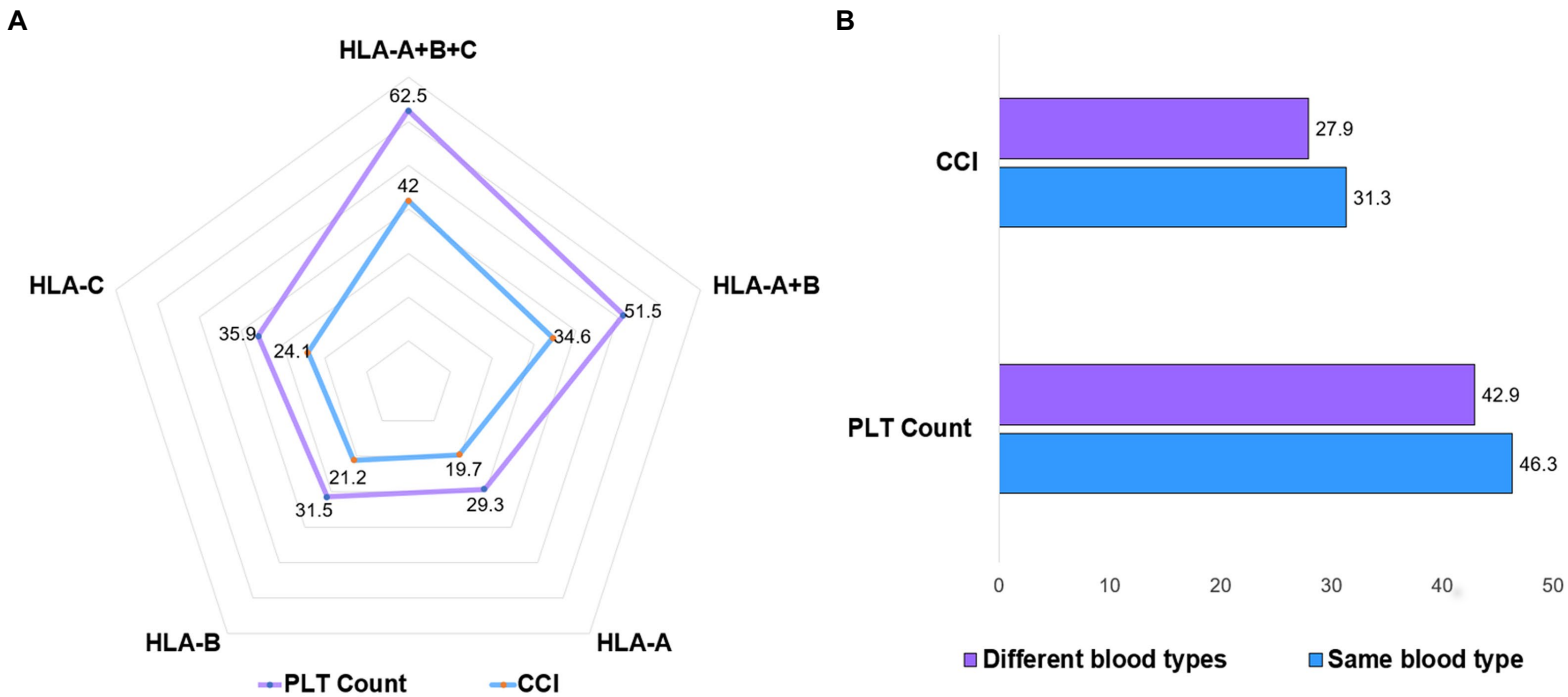
Platelet transfusion effect for HLA and ABO blood types. **(A)** At 24 h after platelet transfusion in treatment group, platelets with the same HLA-A, B, and C had the best transfusion effect, followed by platelets with the same HLA-A and B. **(B)** Platelet transfusions with different blood types did not affect the increase in platelet numbers.

In the treatment group of 31 patients, we analyzed the transfusion effect of platelet donors and recipients with different HLA antigen matching. We found that compared with HLA-A, B, and C, the infusion of HLA-A, B, and C at all three sites was more effective (Figure 4A). However, the different blood types of platelet donors and recipients did not affect the transfusion effect (Figure 4B).

## Discussion

Platelet refractoriness remains a problem in patients with hematological malignancies and severe aplastic anemia because the risk of severe spontaneous or life-threatening bleeding increases significantly when platelet counts fall below 10 × 10^9^/L (12). Because of repeated platelet transfusions in these patients, immune platelets are refractory. According to statistics, the proportion of platelet refractory in patients with bone marrow failure (aplastic anemia, acute leukemia, and myelodysplastic syndrome) is as high as 30–70% (13). Antibodies against class I HLA antigens (A and B alleles) are responsible for most immune-based refractory cases, and patients can use either cross-matched compatible or HLA-matched/compatible platelet units. When trying to select HLA units, it is difficult to find a perfect “4/4” match for a patient’s class IA and IB alleles (14).

In our retrospective study, transfusions were performed using related donor platelets (patients’ parents, siblings, and children). Less bleeding events of WHO grade 1, 2, 3, or 4 occurred in the treatment group than in the comparator group, with a significant decrease in the number of days with bleeding events of WHO grade 1, 2, 3, or 4 and increase time to the first bleeding event of WHO grade 1, 2, 3, or 4. The reduction in bleeding events is attributable to an increase in platelet count and CCI after transfusion. Our results show that platelet transfusion in the treatment group increased number of platelets and CCI at 24 h post-transfusion were more than 4-fold higher than in the comparator group (Table 3), and this advantage persisted even at 72 h post-platelet transfusion.

The main immune factors of platelet refractoriness are class I HLA molecules, platelet-specific glycoprotein, and ABO blood group. These antibodies can bind to cognate antigens on the surface of transfused platelets and clear the transfused platelets from the patient’s circulating blood (15). Among them, the immune factors that lead to platelet refractory are mainly class I HLA molecules. HLA antigens are highly immunogenic. Previous articles reported that the risk of platelet refractoriness in patients with 2 or more pregnancies was 32% (16), repeated transfusions platelets were present in 50% of patients (5, 17). However, in our retrospective study, no platelet refractoriness occurred in the treatment group, but the incidence of platelet refractoriness (24-h CCI < 10) in the comparator group was as high as 68.5%. The main reason for this result is that the HLA-A, B, and C parts of the related donor platelet transfusion are identical. The proportion of all HLA-identical sibs in Chinese siblings reaches 25%, and HLA class I antigens are approximately 80% identical in sibs of parents and children (10). On subgroup analysis in the 31 patients in the treatment group, we found that the platelets with the same HLA-A, B, and C had the best transfusion effect (Figure 4A), which is basically consistent with the previous report (21). In addition, the ABO compatibility of the platelets themselves, as well as the accompanying plasma, must be considered when considering platelet transfusions. Recent data suggest that approximately 31% of platelet transfusions in the United States are severely incompatible (18). Our data also suggest that blood group-matched platelets have better transfusion outcomes (Figure 4B), but the differences in increases in platelet counts are small and not clinically meaningful in terms of bleeding risk.

Although depletion of leukocytes in platelet components has been reported in recent years to prevent the development of HLA alloimmunity (19), or treatment of platelets with ultraviolet beta or gamma irradiation is also an effective method to prevent the development of HLA alloimmunity (20), methods of an epitope-based approach of HLA-matched platelets for transfusion reduced Matching difficulty (21). However, on the one hand, these methods increase the work intensity and increase the cost. On the other hand, these leukocyte inactivating techniques reduce the number of platelets and affect the hemostatic effect (22). Family members can sometimes provide directed platelet units that are a good match, and we also found no differences in platelet directed AEs for family members (Figure 3). It has also been reported that the use of platelets from family members increases the incidence of transfusion-associated graft versus host disease (TA-GVHD) by 2–3 times (23). At present, most medical institutions in China use leukocyte-filtering blood transfusion devices, which can prevent the vast majority of TA-GVHD happened. However, family members who donated platelets as stem cell donors for later allogeneic hematopoietic stem cell transplant patients may produce donor special antibodies (DSAs) that affect stem cell engraftment (24). It has also been shown that the complement inhibitor eculizumab increases CCI in a small number of patients with severe platelet refractoriness (25). However, for patients with severe bleeding tendency and platelet refractoriness, platelet transfusions from related donated by family members may be a more appropriate choice.

## Data availability statement

The original contributions presented in the study are included in the article/supplementary material, further inquiries can be directed to the corresponding author.

## Ethics statement

All procedures involving human participants were in keeping with the ethical standards of the Affiliated Jinhua Hospital, Zhejiang University School of Medicine. All participants in this study offered their written informed consent, and the data were kept anonymous, and the consent was given official approval by the Affiliated Jinhua Hospital, Zhejiang University School of Medicine Ethics Committees. Written informed consent for participation was not required for this study in accordance with the national legislation and the institutional requirements.

## Author contributions

J-CZ and H-XH contributed to the conception and design of this study，had full access to all data in the study, and took responsibility for the integrity of the data and the accuracy of the data analysis. YT and L-HN contributed to writing of the report. H-XH contributed to critical revision of the report. J-CZ contributed to the statistical analysis. All authors contributed to data acquisition, data analysis, or data interpretation, and reviewed and approved the final version.

## Funding

This study was funded by the Research project from Zhejiang Medical and Health Science and Technology Project (No. 2023RC296; Hangzhou, China) and Clinical research Project of Zhejiang Medical Association (No. 2022ZYC-Z39; Hangzhou, China). The study sponsors had no role in the study design, collection, analysis, and interpretation of data, or in the writing of the report.

## Conflict of interest

The authors declare that the research was conducted in the absence of any commercial or financial relationships that could be construed as a potential conflict of interest.

## Publisher’s note

All claims expressed in this article are solely those of the authors and do not necessarily represent those of their affiliated organizations, or those of the publisher, the editors and the reviewers. Any product that may be evaluated in this article, or claim that may be made by its manufacturer, is not guaranteed or endorsed by the publisher.
